# Associations between Medical Conditions and Breast Cancer Risk in Asians: A Nationwide Population-Based Study in Taiwan

**DOI:** 10.1371/journal.pone.0143410

**Published:** 2015-11-25

**Authors:** Shu-Chun Chuang, Guo-Jie Wu, Yen-Shen Lu, Ching-Hung Lin, Chao Agnes Hsiung

**Affiliations:** 1 Institute of Population Health Sciences, National Health Research Institutes, Miaoli, Taiwan; 2 Department of Oncology, National Taiwan University Hospital, Taipei, Taiwan; 3 Department of Internal Medicine, National Taiwan University Hospital, Taipei, Taiwan; School of Medicine, Fu Jen Catholic University, TAIWAN

## Abstract

**Background:**

The breast cancer incidence in Asia is rising. To explore whether the etiology of breast cancer is different from the known risk factors from studies in Western countries, we conducted a nested case-control study using data from the Taiwan National Health Insurance Research Database (NHIRD).

**Methods:**

All medical conditions based on the first three digits of the ICD-9 and a list of medical conditions based on literature review were retrieved for each case and control. The odds ratios (OR) and 95% confidence intervals (CI) of the associations between medical conditions and breast cancer risks were estimated using conditional logistic regression and adjusted for occupation, number of breast cancer screening, and the average number of outpatient visits prior the diagnosis. The associations were also estimated for younger (<50 years old) and older subjects separately.

**Results:**

The analyses included 4,884 breast cancer cases and 19,536 age-matched controls. Prior breast diseases (OR, 95% CI: 2.47, 2.26–2.71), obesity (1.43, 1.04–1.96), endometriosis (1.44, 1.15–1.80), uterine leiomyoma (1.20, 1.03–1.40), hypertensive diseases (1.14, 1.05–1.25), and disorders in lipid metabolism (1.13, 1.04–1.24) were associated with increased breast cancer risk. No heterogeneity was observed between age groups (<50 and ≥50 years old).

**Conclusions:**

In addition to benign breast diseases, obesity, endometriosis, uterine leiomyoma, hypertensive diseases, and disorders of lipid metabolism were associated with a subsequent breast cancer risk.

**Impacts:**

Our results suggest that estrogen related factors may play an important role in breast cancer risks in the Taiwanese female population.

## Introduction

Worldwide, breast cancer was the most common cancer and the most important cause of deaths from cancer in women in 2012 [1], and the incidence is still rising [2]. Particularly in Asia, the annual increase in breast cancer incidences have been reported to be doubled or tripled in the past two decades [3].

Although breast cancer incidence is increasing globally, the pattern differs between Asia and Western countries. The peak age at diagnosis was around 60–70 years in the Western countries, whereas the peak age at diagnosis was about 40–50 years in the Asian populations [2]. It is generally accepted that the etiology of breast cancer is similar worldwide [4], i.e. the reproductive and hormonal factors. The increase in Asia has shown strong birth-cohort effects and the increase may simply reflect the changes in prevalence and composition of risk factors of the breast cancer in the younger generation [5]. It has been noted that the breast cancers in young women are more likely to be “triple-negative” carcinomas (i.e. ER-, PR-, and HER2-) and to be more aggressive than that in the elderly, thus results in higher risks of recurrence or death [6]. However, unlike what is reported in the western countries, young breast cancer patients in Taiwan seem to have higher prevalence of ER+ or PR+ tumors than the older patients [7,8]. These results suggested that westernization may not be the only explanation to the increase in breast cancer incidence.

It has been suggested that cluster of diseases may imply similar etiologies between diseases, common risk factors, or effects of treatment for the prior disease [9]. A prior study had suggested that, in addition to breast cancer, the age-adjusted incidence rates of uterine and ovarian cancers also increased from 1979 to 2007 in Taiwan, and these cancers showed high prevalence of hormone receptor expressions [7]. Other medical conditions, which have been related to breast cancer, were hypothesized to be via estrogen modulation [10], metabolic disruption [11], impaired immune functions or inflammation [12], and medical radiation [13]. Most previous studies on medical conditions and breast cancer risk are based on case-control studies, which may suffer from recall bias, or based on a pre-designed medical list. The Taiwan National Health Insurance (NHI) program covers over 99% Taiwanese population and provides extensive medical information. A National Health Insurance Research Database (NHIRD) was created by National Health Research Institutes (NHRI) for academic research.

The aim of the study was to investigate the etiology of breast cancer by exploring the associations between prior chronic diseases and breast cancer risks using NHIRD.

## Material and Methods

### Source Population

The NHI program has been described previously [14]. Briefly, the program covered over 22 million people in Taiwan, which represented over 99% of the entire population. The Longitudinal Health Insurance Database (LHID)– 2000, 2005, and 2010 is part of the NHIRD and contains claim data of a one-million random sample that covers the beneficiaries registered during 1996–2000, in 2005, and in 2010, respectively. The basic demographic characteristics, e.g. birthday, sex, insured company and area, dates of visits, diagnoses, procedures, and/or prescriptions since 1996 onward were retrieved for each sampled individuals. The ID numbers of the sampled individuals were scrambled. Major diagnoses, such as stroke and acute coronary syndrome, had been validated in previous studies [15,16,17]. To assure the data quality, we set the observation period 2000–2010. The current analyses were based on LHID2000 and LHID2005. The study has been approved by the ethical review board of the NHRI, Taiwan.

### Study Population

Disease and breast cancer status (yes or no) were defined as if a subject ever received care for that specific disease or cancer from any outpatient clinic at least twice or ever been hospitalized due to the specific disease or cancer (based on the major diagnostic code). The earlier date of the visits of the outpatient clinic or the hospitalization was assigned as the date of diagnosis.

**Fig 1:**
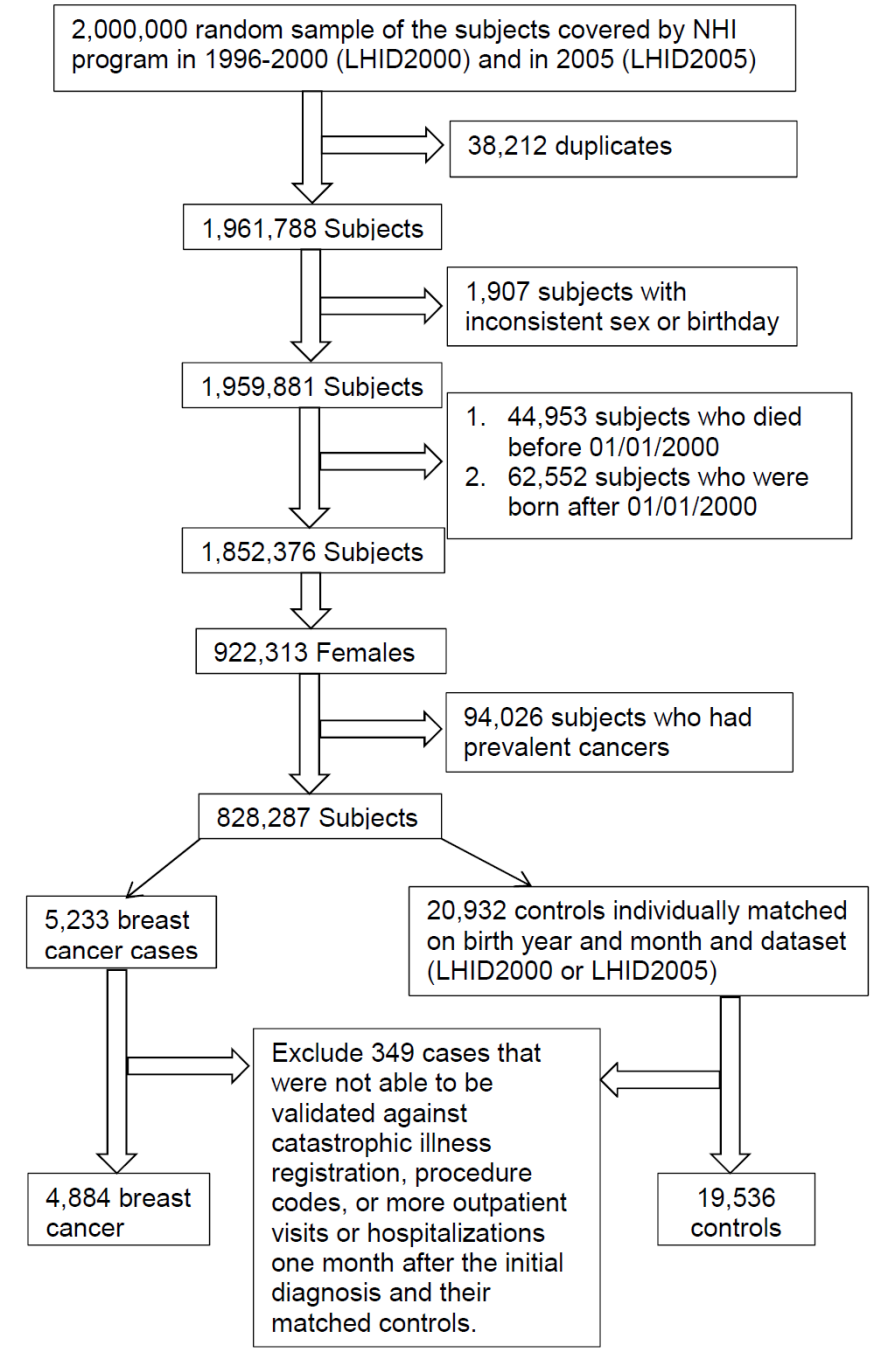
Selection of study population.

Fig 1 shows the procedure of the selection of the study population. 38,212 duplicates were excluded from the combined sample. After carefully screening for the basic insured information, inpatient, and outpatient data files, we also excluded 1,907 subjects with inconsistent sex or birthday. 44,953 subjects who were dead before January 1, 2000 were excluded from the LHID2000 and 62,552 subjects who were born after January 1, 2000 were excluded from both the LHID2000 and LHID2005 because they were not at risk of developing cancer during the entire observation period. Among the remaining 922,313 women, we further excluded those with diagnosis of benign or malignant neoplasms (ICD9: 140–239 or A-code: A08-A17) in 2000 and 2001 (considered as prevalent cases, N = 94,026). Cancers diagnosed after 2002 were defined as newly developed. Among the remaining subjects, we identified 5,233 newly diagnosed breast cancer cases between year 2002 and 2010. Four controls were selected and individually matched to the index case by the birth year and month, and the dataset (LHID2000 or LHID2005). The index dates were defined as the date of the breast cancer diagnosis for the case and the index case’s breast cancer diagnosis date for the controls. The cases were then validated by the catastrophic illness registration (HV), procedure codes, or more outpatient visits or hospitalization one month after the initial diagnosis.

### Statistical Analysis

The breast cancer incidence was calculated from the whole LHID2000 and LHID2005, respectively. Medical histories based on the first three digits of the ICD-9 and the corresponding A-code and aggregated medical conditions based on literature review were retrieved for each case and control (S1 Table). The pre-designed medical condition list includes breast diseases, diseases in hormone modulation, diseases in endocrine system, metabolism related diagnosis, and diseases attributed to common risk factors, e.g., diet and alcohol. Only the disease status prior to the index date was considered. Conditional logistic regression models were used to calculated the odds ratios (OR) and 95% confidence intervals (CI) of the associations between medical conditions and breast cancer risk. The confounding factors included were occupation, number of screening test before the index date (0, 1 and ≥2 times), and the average outpatient visit 6 months prior to the index date. The occupation at the index date was used as a surrogate for socioeconomic status. Occupation was categorized into groups: civil servants, teachers, employees of governmental or private businesses, professionals, and technicians; farmers or fishermen; and low income family supported by social welfare or veterans, people without specific employer, or self-employed people, and the others [18]. The screening variable was retrieved from the outpatient data file with a diagnosed code of V761. The heterogeneity between age groups was estimated by using the *I* ^*2*^ statistics which represents the percentage of total variation contributed by between-study variance [19]. Sensitivity analyses were performed by excluding the casesets that the cases were not validated.

Analyses were performed using SAS 9.3. All tests were two sided and statistical significance was assessed at the level of 0.05.

## Results

Among the 5,233 newly diagnosed breast cancer cases, 2,526 were identified from the LHID2000 and the other 2,707 were identified from the LHID2005 (Table 1). Among these subjects, 4,884 cases can be validated. The calculated age-standardized incidence rates were 42/100,000 in LHID2000 and 44/100,000 in LHID2005 (data not shown). Cases tended to receive one or more screening tests, but there were still more than 90% cases who did not receive any screening before the breast cancer diagnosis. Cases also had higher average outpatient visits than the controls (Table 1).

**Table 1:** Characteristic distribution of the study population.

	LHID 2005					LHID 2000				
	Case		Control			Case		Control		
	N	%	N	%	P^1^	N	%	N	%	p^1^
Total	2514		10056			2370		9480		
**Age**										
<40	325	12.9	1300	12.9	Matched	292	12.3	1,165	12.3	Matched
40–45	342	13.6	1367	13.6		316	13.3	1,271	13.4	
45–50	463	18.4	1849	18.4		440	18.6	1,764	18.6	
50–55	410	16.3	1643	16.3		386	16.3	1,535	16.2	
55–60	289	11.5	1154	11.5		308	13.0	1,232	13.0	
> = 60	685	27.2	2743	27.3		628	26.5	2,513	26.5	
**Index year**										
2002–2004	634	25.2	2536	25.2	Matched	649	27.4	2,596	27.4	Matched
2005–2007	883	35.1	3532	35.1		780	32.9	3,120	32.9	
2008–2010	997	39.7	3988	39.7		941	39.7	3,764	39.7	
**Occupation**										
Civil servants, teachers, governmental or private business employees, professionals, and technicians	1,452	57.8	5,593	55.6	<0.01	1,346	56.8	5,407	57.0	<0.01
Farmers or fishermen	277	11.0	1,608	16.0		284	12.0	1,414	14.9	
Low income families supported by social welfare or veterans, or supported by another family member	785	31.2	2,855	28.4		740	31.2	2,659	28.0	
**Number of screen tests**										
0	9,663	384.4	2,286	22.7	<0.01	9,128	385.1	2,175	22.9	<0.01
1	338	13.4	196	1.9		290	12.2	162	1.7	
> = 2	55	2.2	32	0.3		62	2.6	33	0.3	
**Average ambulatory visit per year**										
≤6	371	14.8	1,755	17.5	<0.01	359	15.1	1,907	20.1	<0.01
6–12	585	23.3	2,434	24.2		574	24.2	2,267	23.9	
12–24	878	34.9	3,450	34.3		835	35.2	3,138	33.1	
>24	680	27.0	2,417	24.0		602	25.4	2,168	22.9	

^1^ Chi-square test or Fisher’s exact test if any number in a cell is less than five.

Medical conditions that presented in at least five cases and were statistically significant associated with breast cancer (p<0.05) in LHID2005 were validated in the LHID2000 and the dataset that combining LHID2000 and LHID2005. Table 2 presents the significant associations in all three datasets. As expected, various forms of breast disease were statistically significant associated with breast cancer. Conversely, general medical examinations (V70) were inversely associated with breast cancer (OR = 0.87, 95% CI = 0.81–0.94).

**Table 2:** Associations that are significant^1^ between prior medical conditions and subsequent breast cancer risk.

ICD9	Disease	LHID2005				LHID2000				LHID2005+LHID2000			
		Control	Case	OR^2^	95% CI	Control	Case	OR^2^	95% CI	Control	Case	OR^2^	95% CI
217	Benign neoplasm of breast	181	136	2.95	(2.33, 3.73)	172	127	2.86	(2.26, 3.64)	353	263	2.90	(2.46, 3.43)
611	Other disorders of breast	613	327	2.20	(1.90, 2.55)	604	352	2.45	(2.11, 2.83)	1,217	679	2.32	(2.09, 2.57)
239	Neoplasms of unspecified nature	80	36	1.68	(1.13, 2.50)	80	40	2.01	(1.36, 2.97)	160	76	1.85	(1.40, 2.44)
523	Gingival and periodontal diseases	5,708	1,568	1.24	(1.12, 1.37)	5,193	1,420	1.18	(1.07, 1.31)	10,901	2,988	1.21	(1.13, 1.30)
521	Diseases of hard tissues of teeth	5,397	1,477	1.20	(1.09, 1.32)	4,927	1,359	1.21	(1.09, 1.33)	10,324	2,836	1.20	(1.12, 1.29)
272	Disorders of lipoid metabolism	1,828	535	1.15	(1.02, 1.30)	1,560	457	1.12	(0.98, 1.27)	3,388	992	1.13	(1.04, 1.24)
789	Other symptoms involving abdomen and pelvis	3,143	774	0.89	(0.80, 0.99)	2,776	682	0.88	(0.79, 0.98)	5,919	1,456	0.88	(0.82, 0.95)
V70	General medical examination	3,248	795	0.86	(0.78, 0.96)	2,904	722	0.88	(0.79, 0.98)	6,152	1,517	0.87	(0.81, 0.94)
724	Other and unspecified disorders of back	3,951	945	0.83	(0.75, 0.92)	3,594	868	0.83	(0.74, 0.92)	7,545	1,813	0.83	(0.77, 0.89)
784	Symptoms involving head and neck	3,395	821	0.86	(0.77, 0.95)	3,006	695	0.78	(0.70, 0.87)	6,401	1,516	0.82	(0.76, 0.88)

^1^ P<0.05 with at least 5 exposed cases.

^2^ Conditional logistic regression based on the criteria that the disease must be 0.5 years prior to the index date. Models were adjusted for occupation, screen tests (never, once, and twice or above), and average ambulatory visit per year

Diseases may be presented in aggregated forms and the codings may depend on the physicians’ practice. Table 3 shows the associations between the selected medical conditions and breast cancer risks. Of note, breast cancer cases had 18.0 (95% CI = 16.4–19.8) times more likely to have breast diseases prior to the breast cancer diagnosis than their matched controls. The risk was greatly reduced if we re-defined a one-year lag time between the breast disease and the breast cancer diagnosis (OR = 2.67, 95% CI = 2.45–2.92). However, the new definition did not change the associations between other medical conditions and breast cancer risks significantly. Further exclude cases who had other newly diagnosed malignant cancers prior their breast cancer diagnosis did not affect the association materially.

**Table 3:** The association between selected medical conditions and breast cancer risks.

	Conditional Logistic Regression				Define disease ≥1 year prior index date				Further exclude cases who had prior cancer history				Further adjust for occupation, screening, and the average outpatient visit per year	
	Control	Case	OR	95% CI	Control	Case	OR	95% CI	Control	Case	OR	95% CI	OR	95% CI
Breast disease	3,131	1,897	18.0	(16.4, 19.8)	1,705	967	2.67	(2.45, 2.92)	1,668	943	2.66	(2.43, 2.91)	2.47	(2.26, 2.71)
Benign neoplasm of breast	411	1,104	13.5	(11.9, 15.3)	353	263	3.12	(2.65, 3.68)	339	253	3.11	(2.63, 3.68)	2.90	(2.46, 3.43)
Disorders of breast	1,602	2,402	11.6	(10.1, 12.7)	1,447	798	2.50	(2.28, 2.75)	1,417	780	2.50	(2.27, 2.75)	2.31	(2.10, 2.55)
Alcohol-related diagnosis	119	37	1.10	(0.67, 1.80)	109	33	1.15	(0.69, 1.92)	108	33	1.22	(0.83, 1.81)	1.12	(0.67, 1.87)
Metabolic disorders (any 3 of the following)	1,258	365	1.19	(1.05, 1.35)	1,080	312	1.18	(1.03, 1.35)	1,043	300	1.17	(1.02, 1.35)	1.09	(0.95, 1.26)
Hypertensive diseases	5,471	1,545	1.26	(1.17, 1.37)	5,065	1,409	1.22	(1.12, 1.32)	4,904	1,357	1.20	(1.11, 1.31)	1.14	(1.05, 1.25)
Diabetes mellitus	2,658	758	1.19	(1.08, 1.30)	2,433	685	1.16	(1.06, 1.28)	2,357	663	1.16	(1.05, 1.28)	1.09	(0.98, 1.20)
Disorders of lipoid metabolism	3,766	1,101	1.26	(1.16, 1.36)	3,388	992	1.25	(1.15, 1.36)	3,273	958	1.25	(1.15, 1.36)	1.13	(1.04, 1.24)
Overweight and obesity	170	59	1.39	(1.03, 1.88)	143	55	1.55	(1.13, 2.11)	139	53	1.53	(1.12, 2.11)	1.43	(1.04, 1.96)
Cholelithiasis or disorders of the gallbladder	717	203	1.14	(0.97, 1.34)	623	176	1.14	(0.96, 1.35)	607	165	1.09	(0.92, 1.30)	1.05	(0.88, 1.25)
Benign neoplasm of ovary	324	105	1.31	(1.05, 1.64)	269	88	1.32	(1.03, 1.69)	263	81	1.24	(0.96, 1.60)	1.21	(0.95, 1.56)
Ovarian dysfunction	204	58	1.14	(0.85, 1.53)	178	55	1.24	(0.91, 1.68)	178	53	1.19	(0.88, 1.63)	1.17	(0.86, 1.59)
Noninflammatory disorders of ovary, fallopian tube, and broad ligament	260	73	1.13	(0.87, 1.47)	238	66	1.11	(0.84, 1.46)	233	63	1.08	(0.82, 1.44)	1.05	(0.79, 1.39)
Disorders of thyroid gland	1,449	431	1.21	(1.08, 1.35)	1,298	379	1.18	(1.05, 1.33)	1,263	360	1.15	(1.02, 1.30)	1.11	(0.98, 1.25)
Endometriosis	370	137	1.51	(1.24, 1.85)	303	111	1.49	(1.19, 1.86)	297	108	1.48	(1.18, 1.85)	1.44	(1.15, 1.80)
Uterine leiomyoma	903	311	1.42	(1.24, 1.63)	758	242	1.30	(1.12, 1.52)	743	236	1.30	(1.11, 1.51)	1.20	(1.03, 1.40)
Disorders of parathyroid gland	35	14	1.60	(0.86, 2.98)	30	9	1.20	(0.57, 2.53)	29	9	1.24	(0.59, 2.62)	1.14	(0.54, 2.43)
Benign neoplasma of rectum and anal canal	37	6	0.65	(0.27, 1.54)	32	5	0.63	(0.24, 1.61)	32	3	0.38	(0.12, 1.23)	0.62	(0.24, 1.61)

Table 4 displays the associations between the selected medical conditions and breast cancer risk by age group. Prior breast diseases were associated with breast cancer risk in both age groups. Obesity, leiomyoma, hypertensive diseases and disorders of lipid metabolism were associated with breast cancer risk only in the older group, but there were no differences in the strength of the associations (p_heterogeneity_ = 0.47 for obesity, p_heterogeneity_ = 0.20 leiomyoma, p_heterogeneity_ = 0.54 for hypertensive diseases and p_heterogeneity_ = 0.39 for disorders of lipid metabolism).

**Table 4:** The association between selected medical conditions and breast cancer risks by age at breast cancer diagnosis.

	<50				≥50				*I* ^*2*^	p for heterogeneity between the two age groups
Name	Control	Case	OR	95% CI	Control	Case	OR	95% CI		
Breast disease	849	498	2.76	(2.42, 3.14)	856	469	2.24	(1.97, 2.55)	79.5%	0.027
Benign neoplasm of breast	180	139	3.24	(2.57, 4.10)	173	124	2.65	(2.08, 3.36)	27.2%	0.241
Disorders of breast	715	418	2.64	(2.30, 3.03)	732	380	2.04	(1.78, 2.35)	84.4%	0.011
Alcohol-related diagnosis	27	11	1.59	(0.79, 3.23)	39	8	0.81	(0.37, 1.75)	16.8%	0.273
Metabolic disorders (any 3 of the following)	78	22	1.00	(0.61, 1.62)	1002	290	1.09	(0.95, 1.27)	0.0%	0.719
Hypertensive diseases	640	180	1.09	(0.91, 1.30)	4425	1229	1.16	(1.05, 1.28)	0.0%	0.535
Diabetes mellitus	292	86	1.10	(0.85, 1.41)	2141	599	1.08	(0.97, 1.21)	0.0%	0.918
Disorders of lipoid metabolism	483	136	1.05	(0.85, 1.28)	2905	856	1.16	(1.05, 1.28)	0.0%	0.386
Overweight and obesity	64	21	1.24	(0.75, 2.04)	79	34	1.59	(1.05, 2.40)	0.0%	0.465
Cholelithiasis or disorders of the gallbladder	154	39	1.00	(0.70, 1.42)	469	137	1.07	(0.88, 1.31)	0.0%	0.734
Benign neoplasm of ovary	200	58	1.08	(0.79, 1.46)	69	30	1.56	(1.00, 2.41)	31.6%	0.227
Ovarian dysfunction	139	40	1.11	(0.77, 1.58)	39	15	1.43	(0.78, 2.62)	0.0%	0.526
Noninflammatory disorders of ovary, fallopian tube, and broad ligament	176	41	0.87	(0.62, 1.23)	62	25	1.58	(0.98, 2.55)	63.1%	0.100
Disorders of thyroid gland	558	164	1.14	(0.95, 1.38)	740	215	1.09	(0.93, 1.28)	0.0%	0.708
Endometriosis	227	82	1.42	(1.10, 1.85)	76	29	1.50	(0.97, 2.33)	0.0%	0.844
Uterine leiomyoma	460	130	1.11	(0.90, 1.37)	298	112	1.37	(1.09, 1.73)	40.5%	0.195
Disorders of parathyroid gland	9	7	3.15	(1.16, 8.51)	21	2	0.34	(0.08, 1.49)	53.5%	0.142
Benign neoplasma of rectum and anal canal	6	2	1.33	(0.27, 6.60)	26	3	0.47	(0.14, 1.56)	0.0%	0.603

## Discussion

In addition to benign breast diseases, which have been recognized as a marker of enhanced risk or a precursor of breast cancer [20], endometriosis and uterine leiomyoma have yield strong association with a subsequent breast cancer. Although the association between diseases and breast cancer risks did not differ by age group, young patients had more uterine leiomyoma (6%) and endometriosis (4%) than the older patients (4% and 1%, respectively).

Estrogen has been implicated in numerous diseases, including cancers of the breast, ovary, colorectal, and endometrial cancers, cardiovascular diseases, osteoporosis, neurodegenerative diseases, insulin resistance, endometriosis, uterine leiomyoma, and obesity [21,22]. A prior study on estrogen-related cancer described increased incidences of breast, uterine, and ovarian cancers and a similar bell-shaped age-specific incidence curve for breast and uterine cancers in Taiwan [7]. Consistent with these results, we also observed strong associations of endometriosis and uterine leiomyoma with breast cancer. On the other hand, estrogen reduces risks of cardiovascular diseases, osteoporosis, and neurodegenerative diseases [23,24]. We observed null association between these diseases and breast cancer (data not shown). Both osteoporosis and neurodegenerative diseases mainly affect the elderly. In our study, we only consider medical conditions prior breast cancer diagnosis. With the improvement of breast cancer survival, it may be interesting to explore the impact of osteoporosis and neurodegenerative disease on breast cancer prognosis.

The association between endometriosis and breast cancer had been reported previously, but the results were inconsistent [25]. However, most of previous studies were conducted in Western countries. A summary result based on a recent review [25] yield a marginal association. A recent publication using the same dataset as ours reported null-association between newly diagnosed endometriosis and future breast cancer risks (HR = 1.15, 95% CI = 0.61–2.15) [26]. The study included 2266 endometriosis cases and 9064 age-matched controls selected from the outpatient dataset. The median age of the study population was 31–40 years old. This study accumulated 69 breast cancer cases in five years (2003–2008). In our study, we examined the association between breast cancer and pre-existing medical conditions; endometriosis is one of the selected medical conditions. The median age of breast cancer diagnosis was 51 years old and it was 46 years old among those who had history of endometriosis. The differences in age distribution and the length of observation period could be the reasons for the discrepancy. Furthermore, our controls were selected from the beneficiaries’ registration data file, which included healthy women who never used the service. That is, the background risk of breast cancer may be different among the two control groups.

We identified three publications [10,27,28] on uterine leiomyoma and breast cancer, but none of these studies reported an association.

Although ovarian diseases share risk factors with breast cancer in endogenous estrogen and reproductive characteristics, most studies failed to observe an association, except one study [29]. However, this study [29] included endometriosis in the ovarian diseases, which may explain the observed associations. Of noted, previous studies suggested that inclusion of women who received oophorectomy may be the reason for the null association between endometriosis or ovarian disease and breast cancer risks [30,31].

Metabolic disorders have been associated with several types of cancer, including the breast cancer [11,32]. Metabolic syndrome and its individual components (except for glucose) were negatively associated with premenopausal breast cancer [11], but positively associated with postmenopausal breast cancer [32]. An earlier NHIRD study, comparing diabetes patients to the age-matched controls, resulted in higher hazard ratio for the older group (≥65 years old, HR = 1.61, 95% CI = 1.45–1.78) [33]. Although diabetes was associated with breast cancer, it loses its association when stratified by age. Nevertheless, the ORs were similar. The null associations could be due to small sample size in subgroups. Adjusting for the average number of outpatient visit decreased the risk estimate, which may suggest the presence of detection bias in diabetic patients. Mutual adjusted for the other metabolic disorders, i.e. hypertensive diseases, diabetes mellitus, disorders of lipoid metabolism, and overweight and obesity, did not change the associations materially (data not shown).

We did not observe an association between thyroid diseases and breast cancer risk. An elevated breast cancer risk is well documented in thyroid cancer patients [34,35,36], particularly in young women [34,35]. A meta-analysis study has reported an association between thyroid diseases and breast cancer [37] although the conclusion is not definitely [38]. It has been suggested that the increased breast cancer risk could be attributed to the iodine ^131^I treatment [27]; however, the evidence was not conclusive, either [39]. Recent evidence of urinary estrogen DNA-adducts suggested that estrogen may act like chemicals to activate the carcinogenic metabolites and initiates both thyroid and breast cancers [40,41].

Most studies on polyps or cholelithiasis and breast cancer were based on the common risk factor theory, e.g. diet. However, except for a few studies [42,43], most studies reported null association, as well as ours. In 2010, the International Agency for Research on Cancer pronounced that “the occurrence of malignant tumors of the oral cavity… and female breast is causally related to consumption of alcoholic beverage” [44]. We examined the association between alcohol-related disorders and breast cancer risk to explore the role of alcohol on breast cancer. However, we did not observe an association between the diseases. This could be because that Taiwanese woman had lower prevalence of heavy episodic drinking (defined as ≥60g pure alcohol at one occasion during the past month, 12% in America, 13% in Europe, and 1.6% in Taiwan) [45,46], which might suggest that alcohol play a minor role in breast cancer in Taiwan.

Other associations, such as gingival and periodontal diseases or unspecified disorders of back are difficult to explain. We cannot rule out the possibility of false findings, although the strengths of associations were similar in both LHID2000 and LHID2005. Nevertheless, it is reasonable that general medical examination (ICD9: V70) was inversely associated with breast cancer risks. It is likely that the use of general medical examination results in early detection of pre-malignant lesions, thus the patient could receive proper treatments before the lesion progresses to a malignant tumor. On the other hand, increased medical surveillance may increase the possibility of detecting a cancer.

Other limitations of the present analyses include that we did not consider the treatment, duration, and the severity of the preceding diseases, as well as the behavior risk factors, in the current analyses. The increased or decreased breast cancer risk could be a result of the treatment of the previous disease or the shared risk factors; therefore, the associations do not imply causality in etiology. Secondly, chance findings could be resulted from multiple comparisons; these results have to be interpreted with causation. Thirdly, having some diseases, e.g. metabolic syndrome, might imply an increased medical surveillance, thus may increase the probability of detecting a breast cancer, and vice versa. These were evident in higher number of screening tests and average ambulatory visit per year in Table 1. This limitation could result in another problem—misclassification—in our study, e.g. the strong association between breast disease and breast cancer. To deal with the problem, we set several lag time to evaluate the impact of misclassification (S2 Table). The association dropped to a reasonable range after a three-month lag time. Another source of misclassification could be due to disease definition. We cannot rule out the possibility of misclassification with our disease definition. In general, increasing the number of claim records is a common strategy to improve accuracy [47,48]. In our dataset, most subjects with medical conditions still had outpatient visits or hospitalization records one month after the initial diagnosis, e.g. >95% for hypertension, diabetes, and disorders of lipid metabolism and >80% for breast diseases, leiomyoma, and endometriosis. However, polyps or benign neoplasm of rectum and anal canal, obesity and overweight and noninflammatory disorders of ovary, fallopian rube, and broad ligament had relatively low validation rate by more outpatient visits or hospitalization records (60%~70%). Because we dealt with cases and controls in the same way, this type of misclassification is usually non-differential between cases and controls. We expected the bias would be toward the null. Fifthly, family history is also a risk factor for breast cancer, particularly among the young patients. It is possible that there may be related subjects in the study population. However, due to the anonymous nature of the database, it is impossible for us to identify the related subjects. Finally, we do not have menopausal status in the dataset; however, matching on age enables us to partially control for this factor.

In general, the use of claim data reduces the possibility of recall bias and assures a relatively completed medical history. Both hypotheses- and data-driven analyses reached similar conclusions. The results from the data-driven analyses may worth for further investigation.

In conclusion, our results suggest that estrogen-related factors may play an important role in breast cancer risks in the Taiwanese female population. It may be worthy of an investigation on the effects of endocrine-disrupting chemicals on estrogen-related diseases, such as endometriosis, uterine leiomyoma, and the related cancers.

## Supporting Information

S1 Table ICD_9 and the A-code of the selected medical conditions (DOCX)

S2 Table The association between selected medical conditions and breast cancer risk by lag time. (DOCX)

## Abbreviations

CI: confidence interval
ICD9: International Classification of Diseases version 9
LHID: Longitudinal Health Insurance Database
NHI: National Health Insurance
NHIRD: National Health Insurance Research Database
OR: odds ratio
NHRI: National Health Research Institutes
