# Supplementary material for: Associations between Medical Conditions and Breast Cancer Risk in Asians: A Nationwide Population-Based Study in Taiwan
Source: PLoS One. 2015 Nov 25;10(11):e0143410. doi: 10.1371/journal.pone.0143410 (PMC4659594; doi:10.1371/journal.pone.0143410)
Supplement: S1 Table — (DOCX) [file pone.0143410.s001.docx]

S1 Table. ICD_9 and the A-code of the selected medical conditions

| **Disease** | **ICD-9-CM** | **A-code** |
| --- | --- | --- |
| Malignant neoplasm of female breast | 174 | A113 |
| **Breast diseases**  Benign neoplasm of breast  Disorders of the breast | 217  610, 611, 612 | A151  A370 |
| **Diseases in hormone modulation** |  |  |
| Benign neoplasm of ovary | 220 | A153 |
| Ovarian dysfunction | 256 |  |
| Noninflammatory disorders of ovary, fallopian tube, and broad ligament  Follicular cyst of ovary  Corpus luteum cyst or hematoma  Other and unspecified ovarian cyst  Acquired atrophy of ovary and fallopian tube  Prolapse or hernia of ovary and fallopian tube | 620.0  620.1  620.2  620.3  620.4 |  |
| Endometriosis | 617 |  |
| Uterine leiomyoma | 218 |  |
| **Diseases in endocrine systems** |  |  |
| Thyroid diseases  Malignant neoplasm of thyroid glands  Disorders of thyroid gland  Simple and unspecified goiter  Nontoxic nodular goiter  Thyrotoxicosis with or without goiter  Congenital hypothyroidism  Acquired hypothyroidism  Thyroiditis  Other disorders of thyroid | 193  240  241  242  243  244  245  246 | A180  A180  A180  A180  A180  A180  A180 |
| Disorders of parathyroid glands | 252 |  |
| **Metabolic related diagnosis** |  |  |
| Diabetes mellitus | 250 | A181 |
| Disorders of lipoid metabolism | 272 |  |
| Overweight and obesity  Localized adiposity | 278.0  278.1 | A183 |
| Hypertensive diseases  Essential hypertension  Hypertensive heart disease  Hypertensive chronic disease  Hypertensive heart and chronic disease  Secondary hypertension | 401  402  403  404  405 | A269  A260  A269  A260  A269 |
| **Diseases attributed to common risk factors: diet and alcohol** |  |  |
| Benign neoplasma of rectum and anal canal  Anal and rectal polyp  Peptic ulcer disease  Colonic polyps | 211.4  569.0  V12.71  V12.72 |  |
| Cholelithiasis  Other disorders of gallbladder | 574  575 | A348  A348 |
| Alcohol-related diagnosis  Alcohol-induced mental disorders  Alcohol dependence syndrome  Alcohol abuse  Alcoholic gastritis  Alcoholic fatty liver  Acute alcoholic hepatitis  Alcoholic cirrhosis of liver  Alcoholic liver damage, unspecified  Toxic effect of alcohol | 291  303  305.0  535.3  571.0  571.1  571.2  571.3  980 | A215  A347  A347  A347  A347 |
