# Supplementary material for: Associations between Medical Conditions and Breast Cancer Risk in Asians: A Nationwide Population-Based Study in Taiwan
Source: PLoS One. 2015 Nov 25;10(11):e0143410. doi: 10.1371/journal.pone.0143410 (PMC4659594; doi:10.1371/journal.pone.0143410)
Supplement: S2 Table — (DOCX) [file pone.0143410.s002.docx]

S2 Table. The association between selected medical conditions and breast cancer risk by lag time

|  | Conditional logistic regression | | | Define disease ≥1 month prior index date | | | Define disease ≥2 months prior index date | | | Define disease ≥3 months prior index date | | |
| --- | --- | --- | --- | --- | --- | --- | --- | --- | --- | --- | --- | --- |
|  | OR | 95% CI | | OR | 95% CI | | OR | 95% CI | | OR | 95% CI | |
| Breast disease | 18.0 | (16.4, | 19.8 ) | 4.38 | (4.04, | 4.75) | 3.54 | (3.26, | 3.84) | 3.40 | (3.12, | 3.69) |
| Benign neoplasm of breast | 13.5 | (11.9, | 15.3) | 5.11 | (4.45, | 5.88) | 4.17 | (3.61, | 4.82) | 3.93 | (3.40, | 4.56) |
| Disorders of breast | 11.6 | (10.1, | 12.7) | 3.85 | (3.53, | 4.19) | 3.23 | (2.96, | 3.53) | 3.15 | (2.88, | 3.44) |
| Alcohol-related diagnosis | 1.10 | (0.67, | 1.80) | 1.10 | (0.67, | 1.80) | 1.10 | (0.67, | 1.80) | 1.10 | (0.67, | 1.80) |
| Metabolic syndrome (any 3 of the following)^1^ | 1.19 | (1.05, | 1.35) | 1.19 | (1.05, | 1.35) | 1.19 | (1.04, | 1.35) | 1.19 | (1.05, | 1.36) |
| Hypertensive diseases | 1.26 | (1.17, | 1.37) | 1.26 | (1.16, | 1.36) | 1.25 | (1.16, | 1.36) | 1.26 | (1.16, | 1.36) |
| Diabetes mellitus | 1.19 | (1.08, | 1.30) | 1.18 | (1.07, | 1.29) | 1.18 | (1.07, | 1.29) | 1.18 | (1.07, | 1.29) |
| Disorders of lipoid metabolism | 1.26 | (1.16, | 1.36) | 1.26 | (1.16, | 1.37) | 1.26 | (1.16, | 1.36) | 1.26 | (1.16, | 1.37) |
| Overweight and obesity | 1.39 | (1.03, | 1.88) | 1.41 | (1.05, | 1.90) | 1.42 | (1.05, | 1.91) | 1.43 | (1.06, | 1.92) |
| Cholelithiasis or disorders of the gallbladder | 1.14 | (0.97, | 1.34) | 1.15 | (0.98, | 1.35) | 1.15 | (0.98, | 1.35) | 1.14 | (0.97, | 1.34) |
| Benign neoplasm of ovary | 1.31 | (1.05, | 1.64) | 1.29 | (1.03, | 1.62) | 1.29 | (1.03, | 1.62) | 1.32 | (1.05, | 1.66) |
| Ovarian dysfunction | 1.14 | (0.85, | 1.53) | 1.13 | (0.84, | 1.52) | 1.14 | (0.85, | 1.54) | 1.15 | (0.86, | 1.55) |
| Noninflammatory disorders of ovary, fallopian tube, and broad ligament | 1.13 | (0.87, | 1.47) | 1.10 | (0.85, | 1.44) | 1.09 | (0.84, | 1.43) | 1.08 | (0.82, | 1.41) |
| Disorders of thyroid gland | 1.21 | (1.08, | 1.35) | 1.19 | (1.06, | 1.34) | 1.17 | (1.04, | 1.31) | 1.18 | (1.05, | 1.33) |
| Endometriosis | 1.51 | (1.24, | 1.85) | 1.51 | (1.23, | 1.85) | 1.50 | (1.22, | 1.84) | 1.50 | (1.22, | 1.84) |
| Uterine leiomyoma | 1.42 | (1.24, | 1.63) | 1.38 | (1.21, | 1.59) | 1.35 | (1.17, | 1.55) | 1.33 | (1.16, | 1.53) |
| Disorders of parathyroid gland | 1.60 | (0.86, | 2.98) | 1.60 | (0.86, | 2.98) | 1.53 | (0.81, | 2.90) | 1.46 | (0.75, | 2.82) |
| Benign neoplasma of rectum and anal canal | 0.65 | (0.27, | 1.54) | 0.65 | (0.27, | 1.54) | 0.65 | (0.27, | 1.54) | 0.56 | (0.22, | 1.42) |

|  | Define disease ≥0.5 years  prior index date | | | Define disease ≥1 year  prior index date | | | Define disease ≥2 years  prior index date | | |
| --- | --- | --- | --- | --- | --- | --- | --- | --- | --- |
|  | OR | 95% CI | | OR | 95% CI | | OR | 95% CI | |
| Breast disease | 3.07 | (2.82, | 3.35) | 2.67 | (2.45, | 2.92) | 2.37 | (2.15, | 2.61) |
| Benign neoplasm of breast | 3.63 | (3.12, | 4.23) | 3.12 | (2.65, | 3.68) | 2.73 | (2.27, | 3.30) |
| Disorders of breast | 2.87 | (2.62, | 3.14) | 2.50 | (2.28, | 2.75) | 2.22 | (2.01, | 2.46) |
| Alcohol-related diagnosis | 1.14 | (0.70, | 1.88) | 1.15 | (0.69, | 1.92) | 1.05 | (0.61, | 1.82) |
| Metabolic syndrome (any 3 of the following)^1^ | 1.17 | (1.02, | 1.33) | 1.18 | (1.03, | 1.35) | 1.20 | (1.03, | 1.39) |
| Hypertensive diseases | 1.24 | (1.15, | 1.35) | 1.22 | (1.12, | 1.32) | 1.18 | (1.08, | 1.28) |
| Diabetes mellitus | 1.17 | (1.07, | 1.29) | 1.16 | (1.06, | 1.28) | 1.16 | (1.05, | 1.28) |
| Disorders of lipoid metabolism | 1.26 | (1.16, | 1.37) | 1.25 | (1.15, | 1.36) | 1.26 | (1.15, | 1.38) |
| Overweight and obesity | 1.44 | (1.06, | 1.94) | 1.55 | (1.13, | 2.11) | 1.61 | (1.15, | 2.27) |
| Cholelithiasis or disorders of the gallbladder | 1.11 | (0.94, | 1.32) | 1.14 | (0.96, | 1.35) | 1.08 | (0.90, | 1.30) |
| Benign neoplasm of ovary | 1.31 | (1.04, | 1.66) | 1.32 | (1.03, | 1.69) | 1.28 | (0.97, | 1.69) |
| Ovarian dysfunction | 1.20 | (0.89, | 1.61) | 1.24 | (0.91, | 1.68) | 1.24 | (0.89, | 1.71) |
| Noninflammatory disorders of ovary, fallopian tube, and broad ligament | 1.10 | (0.84, | 1.44) | 1.11 | (0.84, | 1.46) | 1.11 | (0.82, | 1.51) |
| Disorders of thyroid gland | 1.16 | (1.04, | 1.31) | 1.18 | (1.05, | 1.33) | 1.18 | (1.04, | 1.34) |
| Endometriosis | 1.54 | (1.25, | 1.90) | 1.49 | (1.19, | 1.86) | 1.48 | (1.17, | 1.88) |
| Uterine leiomyoma | 1.32 | (1.14, | 1.52) | 1.30 | (1.12, | 1.52) | 1.28 | (1.08, | 1.52) |
| Disorders of parathyroid gland | 1.38 | (0.69, | 2.73) | 1.20 | (0.57, | 2.53) | 0.91 | (0.35, | 2.41) |
| Benign neoplasma of rectum and anal canal | 0.63 | (0.24, | 1.61) | 0.63 | (0.24, | 1.61) | 0.67 | (0.23, | 1.92) |
